# Assessing the feasibility of HPV screening for cervical cancer in pregnant women in Ethiopia

**DOI:** 10.1038/s41598-025-15957-y

**Published:** 2025-08-28

**Authors:** Selamawit Mekuria, Nahom Assiged, Habtamu Biazin, Christer Borgfeldt, Tamrat Abebe, Adane Mihret, Ola Forslund, Mats Jerkeman

**Affiliations:** 1 Department of Medical Oncology, Lund University, 221 00 Lund, Sweden; 2 Armauer Hansen Research Institiute, Addis Ababa, Ethiopia; 3 Department of Obstetrics and Gynecology, Linköping University, Linköping, Sweden; 4 Department of Biomedical and Clinical Sciences, Linköping University, Linköping, Sweden; 5 Department of Microbiology, Addis Ababa University, Addis Ababa, Ethiopia; 6 Department of Translational Medicine, Lund University, Malmö, Sweden

**Keywords:** Pregnancy, HPV self-sampling, Cervical cancer screening, Population screening, Cancer screening

## Abstract

Pregnant women have historically and are currently being excluded from cervical cancer screening in most low and middle-income countries (LMICs). The aim of this study was to assess the feasibility and outcomes of including pregnant women in a HPV self-sampling-based screening program in Ethiopia. Pregnant women, recruited from a previously established cohort, were included. They answered a questionnaire and provided HPV self-samples. If the woman was HR-HPV positive, she underwent triage with VIA with or without Iodine. If positive in triage, the woman was re-scheduled after delivery for a new exam. Primary outcome was screening participation. The participation rate of pregnant women was 92.1% (117/127) (95% CI 86.0–96.1%). They had the same knowledge about cervical cancer and acceptance rate to the study as their non-pregnant peers. Pregnant women had less history of previous screening (p = 0.08). The HPV prevalence was 25.4% (29/114) in self-samples. 93.1% (27/29) attended follow-up, where only 11 had not delivered, and 54.6% (6/11) had detectable HPV infection in their cervical samples. Including pregnant women in HPV self-sampling-based screening is feasible and highly accepted. The findings support integrating pregnant women into cervical cancer screening programs in to enhance prevention and early detection efforts.

Clinical trials ID: NCT05125380.

## Introduction

Pregnant women have historically been excluded from cervical cancer screening in low and middle-income countries (LMICs)^1,2^. Whereas in some high-income countries (HIC), such as Sweden, the first antenatal visit is often used to catch those who have not participated in the regular screening calls^3^. One major reason for this historical difference, has been the previous recommendation of a single-visit approach in LMICs. This meant visual inspection with acetic acid(VIA), treating all positive lesions directly, which in pregnant women is not possible^4^. The aim of cervical cancer screening in pregnancy is to exclude cancer, not treating pre-cancerous lesions^5^. Moreover, fear of bleeding or abortion among women and difficulties assessing a congested cervix by health professionals, are other reasons why screening of pregnant women has been overlooked^5^.

However, even where cytology has been the screening standard, such as in the United Kingdom, the recommendation from the National Health Service (NHS), is to delay the screening test until at least 3 months post-delivery^6^. This is done despite cervical cancer being one of the most common cancers in pregnancy^7^. Moreover, and most importantly, for many women in LMICs, pregnancy may be the only time they visit a health facility, as both antenatal visits and births at health facilities are increasing with ongoing efforts^8^. A majority of pregnant women are now attending at least one antenatal visit^9^, and in Ethiopia the antenatal coverage with 4+ visits was increasing before the covid-19 pandemic (from 16% in 2005, to 35% in 2016)^10^. Ethiopia introduced a national cervical cancer screening program through VIA in 2015^11^. Despite this program, screening coverage is less than 10%^11–13^.

The experience of including pregnant women in cervical screening programs in low-resource settings is scarce. One study from Nigeria evaluated the prevalence and progression/regression of cervical intraepithelial neoplasia (CIN) in pregnant women by using cytology^14^. They recruited women from antenatal clinics and found a 6% prevalence of CIN and that about half regressed postpartum. However, due to its logistical and quality challenges, cytology is not primarily recommended for LMICs^15^. Despite this, pap smear along with HPV diagnostic was offered in India at an antenatal clinic^16^. The authors found it feasible despite some difficulty of visualising the cervix, and the participation rate was high (98.5%). They acknowledged that more pregnant women are now seeking antenatal care and are giving birth in medical facilities. There, the antenatal clinic is an opportunity to screen women who would not otherwise participate in preventative healthcare^16^.

The number of pregnancies (both intended and unintended) in the developing world is high, 190 million yearly^17^. Even when assuming two thirds would not fall within WHO recommended screening age, the number of pregnancies that might not even be continued (because of miscarriage and abortion), is high. This is a very large population of women who are presently being excluded from cervical cancer screening. As the age of pregnant women in LMICs, similar to the rest of the world, increases, the risk of non-communicable diseases, such as cervical cancer will increase in this group^18^.

To reach the World Health Organisation (WHO) 90,70,90 goal for elimination of cervical cancer, screening opportunities offered for women living in LMICs need to be broadened.

We created a nested cohort that was part of a larger randomised controlled trial (RCT) in Adama, Ethiopia between 2021 and 2023^19^. The aim of this cohort study is to assess the feasibility and the outcome of the pregnant women participating in an HPV self-sampling based screening program.

## Method

### Study design

This observational study was based on a subpopulation of individuals, participating in a larger RCT^19^, which in turn was nested into a cohort previously established through a joint venture between Armauer Hansen Research Institute, Ethiopia and Lund University, Sweden (ALURS). The ALURS cohort was initiated in 2014 by including women attending antenatal clinics in Adama, Ethiopia^20^.

### Study population

Starting in December 2021, all women from the ALURS cohort were invited to participate in a RCT^19^ evaluating a cervical cancer screening programme based on HPV DNA self-sampling, using VIA with or without Iodine for triage. For this observational study, women were selected from the RCT based on being pregnant at inclusion. Exclusion criteria were ongoing cervical cancer treatment and age under 18 years.

### Data collection

Women were scheduled by phone to visit their local health centre. An animation, created by the research team together with a media company in Ethiopia, shown on a tablet, was used to educate the women on cervical cancer screening. If the woman agreed to participate, she was asked to answer a questionnaire. The questionnaire was interview-led and covered mainly screening history and presence of current clinical symptoms (Supplemental material). Study data was collected and managed using RedCap electronic data capture tools hosted by Lund University^21,22^. Demographic data was mainly collected though the ALURS cohort database. All women were after the interview asked to participate in the screening part of the study.

**Fig. 1:**
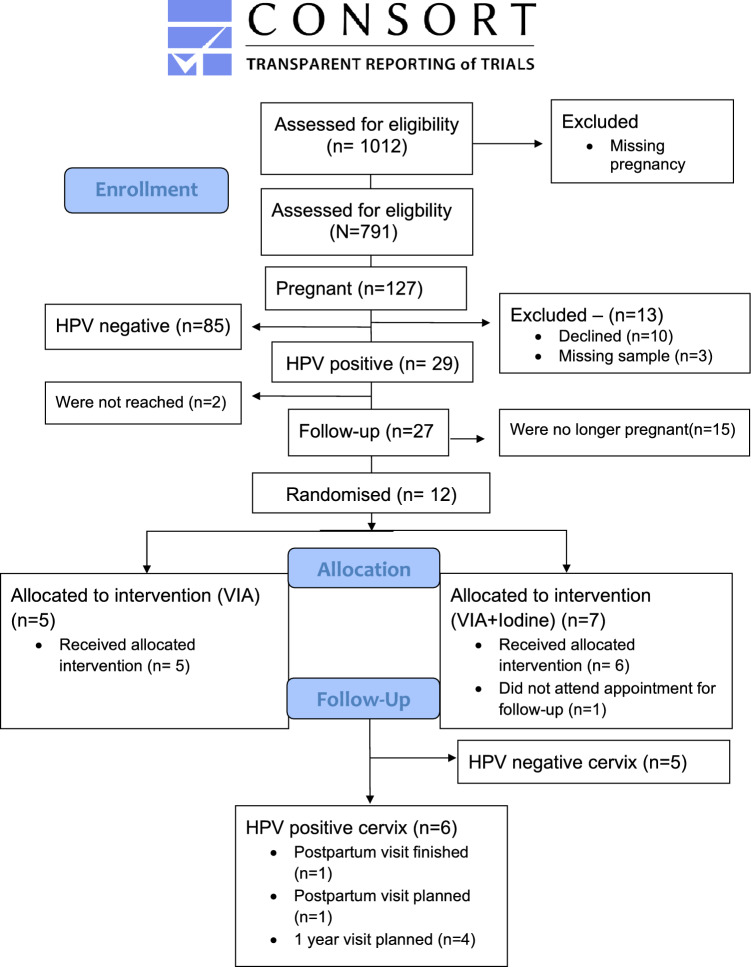
Consort flow diagram.

All accepting women received two eNAT^**®**^ Swabs (Copan, USA) and visited the bathroom at the health-centre to take their own vaginal sample. All women testing HPV DNA positive by the use of Anyplex™ II HPV HR Detection kit (Seegene, Inc; Korea) were planned to be followed-up at the midwife-led clinic. For the larger RCT^19^, all participants were stratified according to age and pregnancy status, then randomised to VIA (control) with or without Lugol’s Iodine (intervention), using RedCap^21,22^. Participants were blinded to the intervention arm. The consort diagram from the RCT has been adjusted to focus on the pregnant subgroup (Fig. 1).

At the follow-up appointment, the midwife explained once again the difference between HPV, precancer and cancer to the woman, as this has been explored in the animation at inclusion. If suspicion of a pre-cancerous lesion existed during the visit, the woman was informed that she would be re-appointed after delivery. The aim of the follow-up for pregnant women was to exclude the suspicion of cancer through triage, not to treat the woman with pre-cancerous lesions.

A gynaecological exam was then performed, and the midwife took an HPV DNA test using eNAT^**®**^ swab (Copan, USA) from the cervix. The midwife was blinded to the cervical HPV result as this was reported after analysis at a central laboratory. Women were examined with acetic acid or acetic acid with lugol’s iodine applied to the cervical surface for the triage test^19^. No biopsy was taken unless there was a suspicion of cancer. If positive in the triage test^19^ and if HPV DNA positive in the cervix, the women were re-appointed at least two months after delivery for a new evaluation at the midwife-led clinic.

### Variables

Primary outcome: screening participation rate in for pregnant women.

Secondary outcomes: Prior knowledge of cervical cancer, previous screening history, outcome of screening.

### Methods of analysis

STATAcorp was used for statistical analysis^23^. Descriptive statistics were used to demonstrate the characteristics of the participants. For comparison between pregnant and non-pregnant women, Mann–Whitney test was used for continuous variables, and Fisher’s exact test for smaller proportions. The Chi^2^ was used to compare parity. Logistic regression was used to calculate odds ratio. As there were missing data for some variables, the numerator may differ in the calculations. Feasibility was evaluated based on participation, outcome of screening and practicality of the intervention.

## Results

At the initiation of the study, 16.1% (127/791) of the women were pregnant (Fig. 1). In Table 1, demographics and clinical characteristics are presented. Median age was 31 years, with a range between 24 and 45 years. Most (93.7%) were married and 15.7% (N = 20) were multipara (para three and above). HIV prevalence (self-reported) was 7.0% (N = 9).

**Table 1:** Participants’ demographics based on their inclusion criteria, pregnancy.

	Pregnancy status			p-value
	Pregnant N = 127 (16.1%)	Non-pregnant N = 664 (83.9%)	Unknown/missing N = 221	
Median age (range)	31 (24–45)	33 (22–47)	32 (25–47)	0.0002
Marital status				0.1
Single	6 (4.7%)	13 (2.0%)	8 (3.6%)	
Married	119 (93.7%)	646 (97.6%)	212 (95.9%)	
Widowed	0	2 (0.3%)	0	
Divorced	2 (1.6%)	1 (0.2%)	0	
Educational history				0.4
Illiterate	11 (8.7%)	75 (11.3%)	33 (14.9%	
< 6 grade	26 (20.5%)	140 (21.1%)	36 (16.3%)	
6–12 grade	77 (60.6%)	368 (55.5%)	130 (58.8%)	
Higher education	13 (10.2%)	80 (12.1%)	21 (9.5%)	
Parity				0.4
1	46 (36.2%)	213 (32.1%)	71 (32.1%)	
2	61 (48.0%)	262 (39.5%)	85 (38.5%)	
> 2	20 (15.7%)	189 (28.5%)	65 (29.4%)	
Residence				1.0
Rural	1 (0.8%)	10 (1.5%)	3 (1.4%)	
Urban	125 (98.4%)	647 (97.4%)	215 (97.2%)	
Missing	1(0.8%)	7 (1.1%)	3(1.4)	
HIV status				0.1
Negative	116 (91.3%)	603 (90.8%)	187 (84.0%)	
Positive	9 (7.1%)	52 (7.8%)	28 (12.7%)	
Missing	2(1.6%)	9(1.4%)	6(2.7%)	
Prior knowledge of cervical cancer screening	85 (66.9%)	420 (63.3%)	143 (64.7%)	0.4
No prior knowledge of cervical cancer screening	42 (33.1%)	244 (36.8%)	77 (34.8%)	
Previously been screened for cervical cancer	9/85 (10.6%)	79/423 (18.8%)	26/143 (18.7%)	0.08

The participation rate of pregnant women in our study was 92.1% (117/127) (95% CI 86.0–96.1%) in comparison to 91.1% (605/664) (95% CI 88.7–93.2%) among those not pregnant. The odds of being pregnant and giving consent to screening was the same as for non-pregnant women (OR 1.0, p = 1.0). Increasing age was associated with a slight decrease in the odds of giving consent (p = 0.9).

Most pregnant women had previous knowledge of cervical cancer screening, similar to non-pregnant women. Numerically fewer pregnant women had a history of prior screening, 9/85 (10.6%) vs 79/423 (18.8%), p = 0.08.

The HPV positivity rate was 22.8% (29/127) (95% CI 14.3–29.9%) among pregnant, and in non-pregnant women 22.4% (127/878) (95% CI 19.7–25.3%). HPV genotype 35 and 56 were the most common types found (Table 2).

**Table 2:** HPV genotypes present at inclusion for pregnant women.

HPV genotypes	16	18	31	33	35	39	51	52	56	58	66	68
Frequency	2	2	1	1	6	1	2	2	7	2	2	1
Percentage	6.9	6.9	3.5	3.5	20.7	3.5	6.9	6.9	24.1	6.9	6.9	3.5

At the follow-up visit, 11 women were pregnant (78.6%) (Table 3). The time to follow-up was highly variable and was incompletely documented. A total of 27.3% (3/11) pregnant women were triage positive, and according to protocol planned to be booked after delivery. No suspicion of cancer was identified among the pregnant women. In the second HPV test, HPV was present in N = 6/11 (54.6%) and N = 52/112(46.4%) of pregnant and non-pregnant women who previously presented with a positive HPV vaginal sample, respectively (p = 0.8) (Table 3).

**Table 3:** Data collected at follow-up of pregnant women compared to non-pregnant women.

	Pregnancy status		p-value
	Pregnant	Non-pregnant	
Triage negative	8 (73.7%)	95 (83.3%)	0.4
Triage positive	3 (27.3%)	19 (16.7%)	
HPV positivity in cervix	6 (54.6%)	52 (46.4%)	0.8
Total women at follow-up (n)	11	112	

Only one woman at follow-up was triage positive and had a positive cervical HPV sample (HPV type 35). She was re-booked after delivery for a new VIA, which she had previously been randomised to. At this postpartum follow-up the triage test was negative. The HPV cervical test was positive, with the same HPV type 35, as in her self-sample.

All women, regardless of pregnancy status with a persisting/cervical HPV positive test, were or will be re-booked one year after for a new HPV self-sample at the local health centre.

## Discussion

Pregnant women constituted a minority (16%) of participants in the larger randomized controlled trial (RCT) into which this study was nested^19^.This might lead some to question the necessity of including pregnant women in cervical cancer screening initiatives. However, as the age distribution indicates, more than half of the pregnant participants were within the World Health Organization’s recommended screening age (over 30 years). Furthermore, knowledge about cervical cancer screening was comparable between pregnant and non-pregnant women. Although not statistically significant, there was a numerical trend suggesting that pregnancy was associated with a history of non-participation in cervical cancer screening, despite equivalent levels of awareness. Notably, pregnancy did not appear to influence a woman’s willingness to participate in screening within our study. The established convenience of self-sampling methods likely contributes to this willingness, regardless of pregnancy status^24^.

The HPV prevalence rate did not differ from non-pregnant women. It is known that pregnancy does not alter HPV or cervical disease progression^25–29^. However, in a recent review of the literature, four of the six of studies included demonstrated a higher HPV prevalence rate in pregnancy than in in non-pregnant women^30^. The authors argue that this may have to do with differences in between the two groups that are unrelated to pregnancy, which could have led to a higher detection rate.

At the time of follow-up, most women who had been pregnant at the time of inclusion had already delivered. Restricting cervical cancer screening to non-pregnant periods presents logistical challenges and represents a missed opportunity for early detection. Pregnancies may end prematurely, either spontaneously or through medical intervention, and there is no guarantee that healthcare providers will encounter the woman again before a subsequent pregnancy. This concern is underscored by global statistics indicating that at least 15% of all pregnancies result in miscarriage^31^, and on top of this, 10% of all births are delivered prematurely, a majority of which occur in Sub-Saharan Africa and Southern Asia^32,33^.

By screening everyone regardless of pregnancy status, the 15 women who we reached at follow-up and were no longer pregnant, could follow the regular RCT protocol including biopsies^19^. We contend that HPV self-sampling is safe and does not discriminate based on current reproductive status.

In contrast to the findings by Sudhakran et al.^16^, where only 36.4% had prior knowledge of cervical cancer screening, most women in our study had heard about this subject before. One reason may be that the Ethiopian ministry of health has conducted campaigns through different outlets for a few years. TV and radio were the main source of knowledge regarding cervical cancer screening in a qualitative study from Eastern Ethiopia^34^.

Sudhakran et al. used the antenatal visit to increase the initial low level of awareness among the participants^16^. Thereafter they asked the women to join the screening with cytology and HPV. The need for an examination room and trained human resources, makes this approach difficult to scale up in a low-resource setting. They suggested HPV self-sampling as an alternative primary screening method to reduce the number of physical out-patient examinations. We agree with them.

Both Sudhakran et al. and Bakari et al. carried out their studies at an antenatal clinic^14,16^. In a recent Ghanaian study, women were included from both ante- and postnatal clinics, but the screening occurred at a separate cervical cancer screening unit^35^. We know that globally there is an increase in antepartum care visits, and that most women in LMICs now receive at least one visit with a health-provider during their pregnancy^36^. Our study screened women at health centres, as part of a larger RCT. Therefore, a limitation in our study is that we cannot evaluate the use of antenatal clinics as a platform for information and screening of cervical cancer. However, lessons from studies such as above, and answers from our study reveal that women are willing to be screened even during pregnancy, which is important when considering the feasibility of offering HPV self-sampling. This leads us to consider that it is an untapped possibility to reach the WHO goal of 70% screening coverage^15^.

In our study we did not see any significant difference in triage results or cervical HPV presence among pregnant and non-pregnant women. However, this may be due to the small number of participants, which is another limitation of this study. The follow-up worked well but needs the experience of a healthcare professional who is used to examining the pregnant cervix. Furthermore, no dysplasia nor cancer were found in our pregnant cohort. Nevertheless, previous knowledge informs us that cervical dysplasia does not progress more rapidly during pregnancy, but rather that it is more common for women to heal from their dysplasia postpartum^37^. Accordingly, prior to the follow-up examination, participants were informed that the purpose of the visit was solely to rule out malignancy and that the procedure was safe. No concerns were expressed by the patients; on the contrary, several informally emphasized the importance of undergoing examination precisely because they were pregnant.

To follow-up a woman postpartum, which only occurred in one of our participants, the logistics must been in place. Postnatal visits have generally low coverage globally and in particular in LMICs, despite the fact that 30% of maternal deaths occur in the postpartum period^38^. WHO recommends three visits, the last one being 6 weeks postpartum. It may be an opportunity to follow those that have tested HPV positive during pregnancy, and not had their follow-up completed. WHO guidelines on antenatal/postnatal and family planning services could consider including cervical cancer screening with HPV self-sampling as part of the visit. Experience with integrating health-services for the mother with for example the child’s immunization in the postpartum era, or family planning services^39,40^ exists from before. To similarly integrate cervical cancer screening may result in several improved public health outcomes, as human resources are used more efficiently. It will however require planning and continuous education for the staff, and the need for more colleagues who can deliver both reproductive health related care and cervical cancer screening.

## Conclusion

This is to the best of our knowledge, the first study to include pregnant women from a low-resource setting and demonstrate that it is feasible and safe for them to be part of a HPV self-sampling-based cervical cancer screening program. Pregnant women have the same wish to be screened as non-pregnant women. This study allowed 127 women who otherwise would not have been screened, to participate and decrease their risk for cervical cancer. We suggest including pregnant women in efforts to increase screening participation in LMICs. Future studies need to evaluate an implementation protocol integrating HPV self-sampling-based screening into antenatal and postnatal clinics.

## Supplementary Information

Supplementary Information.

## Data Availability

The datasets used and/or analysed during the current study are available from the corresponding author on reasonable request.

## Abbreviations

LMICs: Low-and-middle-income countries
HIC: High-income countries
RCT: Randomised controlled trial
ALURS: Adama Lund Research Site
