# Supplementary material for: Assessing the feasibility of HPV screening for cervical cancer in pregnant women in Ethiopia
Source: Sci Rep. 2025 Aug 28;15:31771. doi: 10.1038/s41598-025-15957-y (PMC12394452; doi:10.1038/s41598-025-15957-y)
Supplement: Supplementary file 1 — Supplementary Information. [file 41598_2025_15957_MOESM1_ESM.pdf]

Cc And Sti Screening History

Study code	______________________
Date of HPV screening	______________________
Have you ever heard of cervical cancer screening before?	◯ Yes<br>◯ No
Have you ever been screened for cervical cancer?	◯ Yes<br>◯ No
Have you undergone treatment as a result of your cervical cancer screening?	◯ Yes, with freezing technique<br>◯ Yes, with hysterectomy<br>◯ No
Have you ever been treated for genital infection?	◯ Yes<br>◯ No
Do you have any of the following symptoms right now?	☐ lower abdominal pain<br>☐ vaginal discharge<br>☐ abnormal vaginal bleeding<br>☐ none

If the woman described any physical symptoms, she should after consent be counselled to seek medical help with a gynaecologist in addition to the HPV sample.

Is the woman eligible for the HPV study (age above 18, no hysterectomy)?	◯ Yes<br>◯ No
Consent for the HPV study?	◯ Yes<br>◯ No
Current age	◯ Less than 30<br>◯ 30 and older
Is the woman pregnant?	◯ Yes<br>◯ No
When is your due date?	______________________

Cc And Sti Result

Study code ______________________

Microbiology lab

Self-collected HPV result

- ◯ positive
- ◯ negative

HPV genotypes

- ☐ 16
- ☐ 31
- ☐ 35
- ☐ 45
- ☐ 52
- ☐ 58
- ☐ 66
- ☐ 18
- ☐ 33
- ☐ 39
- ☐ 51
- ☐ 56
- ☐ 59
- ☐ 68

STI result

- ☐ negative
- ☐ pos chlamydia
- ☐ pos gonnorhea
- ☐ pos mycoplasma hominis
- ☐ pos mycoplasma genitalium
- ☐ pos trichomonas
- ☐ pos ureaplasma urealytikum
- ☐ pos ureaplasma parvum

Adama Health Center

STI treatment given

- ◯ no
- ◯ yes only to the woman
- ◯ yes, to the woman and her partner

Cc And Sti Follow Up

Study code

____

Date of CC and STI follow up

____
(Automatically filled)

Date of CC and STI follow up determined from data entry log

____
(Automatically filled)

Randomization (done at the office)

Current age

◯ Less than 30
◯ 30 and older

Is the woman pregnant?

◯ Yes
◯ No

When is your due date?

____

Randomization VIA/VILI

◯ VIA
◯ VIA and VILI

If not randomized, which follow-up was allocated?

◯ VIA
◯ VIA and VILI

Cc And Sti Clinic Follow Up

Study code

____

VIA clinic - follow-up

Date of CC and STI clinic follow up

____
(Automatically filled)

Information about VIA/VILI, biopsy and follow up given
- ◯ Yes
- ◯ No

HPV test taken from cervix
- ◯ Yes
- ◯ No

VIA/VILI result
- ◯ negative
- ◯ positive
- ◯ Suspicion of cancer
- ◯ inconclusive

Follow-up with gynaecologist should be planned

Cervical biopsy taken
- ◯ Yes
- ◯ No

Treatment of lesion
- ◯ no, bc of pregnancy
- ◯ yes with cryotherapy
- ◯ yes, with thermal ablation

Pathology and Microbiology results

Cervical biopsy results

____

HPV cervix result
- ◯ Pos
- ◯ Neg

HPV cervical genotypes present
- ☐ 16
- ☐ 18
- ☐ 31
- ☐ 33
- ☐ 35
- ☐ 39
- ☐ 45
- ☐ 51
- ☐ 52
- ☐ 56
- ☐ 58
- ☐ 59
- ☐ 66
- ☐ 68

Are you HIV positive?	◯ Yes<br>◯ No<br>◯ Don't know<br>◯ Don't want to disclose
If HIV-positive, when did you recive the diagnosis, and when did you start treatment?	________________________________
